# The effects of combined exposure of solvents and noise on auditory function – A systematic review and meta-analysis

**DOI:** 10.4102/sajcd.v66i1.568

**Published:** 2019-05-09

**Authors:** Faatima Nakhooda, Benn Sartorius, Samantha M. Govender

**Affiliations:** 1 Discipline of Audiology, School of Health Sciences, University of KwaZulu-Natal, Durban, South Africa; 2 School of Nursing and Public Health, University of KwaZulu-Natal, Durban, South Africa

**Keywords:** Solvents, solvent induced hearing Loss, SIHL, ototoxicity, noise Induced hearing loss

## Abstract

**Background:**

Chemical substances can negatively affect the auditory system. Chemical substances alone or combined with high-level noise have recently become a major concern as a cause of occupational hearing loss.

**Objective:**

To assess the combined effect of solvents and noise versus solvents only, or noise only, on the auditory function of workers.

**Method:**

Published articles which included noise and/or solvent exposure or combined effects of solvents and noise, studies conducted on human beings only and the use of audiological tests on participants.

**Results:**

Thirteen papers were eligible for inclusion. The participants’ ages ranged from 18 to 68 years. Results revealed that 24.5% presented with hearing loss as a result of noise exposure only; 18% presented with hearing loss owing to solvent exposure only; and a total of 43.3% presented with hearing loss owing to combined noise and solvent exposure. Furthermore, the prevalence of hearing loss in the noise and solvent group was significantly (*p* < 0.001) higher than the other groups in 10 out of the 13 studies analysed, with a pooled odds ratio (OR) of 2.754. Of the 178 participants (total of all participants exposed to solvents), a total of 32 participants presented with auditory pathology as a result of exposure to solvents only. There was a significantly higher pooled odds of hearing loss in noise and solvent-exposed group compared to solvent-only group (pooled *OR* = 2.15, 95% CI: 1.24–3.72, *p* = 0.006).

**Conclusion:**

The findings revealed significantly higher odds of acquiring hearing loss when workers were exposed to a combination of solvents and noise as opposed to solvents only, motivating for its inclusion into hearing conservation programmes.

## Introduction

The body of evidence has been growing regarding not only the effects of chemical substances on the auditory system but also on the combined auditory effects of chemicals and noise on hearing (Johnson & Morata, 2010; Mirzaei & Ansari-Moghaddam, 2012). Chemical substances alone, or combined with high-level noise, have recently become a major concern as a cause of occupational hearing loss (Johnson & Morata, 2010). Previously, noise was believed to be the only cause of hearing loss among workers in occupational settings; however, recent studies have revealed that chemical substances which include solvents can also have ototoxic effects on the auditory system thus resulting in hearing loss (Mirzaei & Ansari-Moghaddam, 2012). Solvents are liquids that are used to dissolve substances, are colourless, have strong odours and have been noted in recent literature to induce auditory pathology. For the purpose of the review, the term ‘Solvent-Induced Hearing Loss’ (SIHL) will be used, as solvents are ototoxic agents that cause hearing loss (Fuente & McPherson, 2006, 2012).

Workers within occupational settings are exposed to various work-related substances that may be hazardous to hearing such as asphyxiants, pesticides, metals and solvents. Morata and Little (2002) categorised solvents into two categories, high-priority solvents such as toluene, xylene, styrene, n-hexane and trichloroethylene, and low-priority solvents such as benzene (Johnson & Morata, 2010; Mirzaei & Ansari-Moghaddam, 2012; Morata & Little, 2002). Both high- and low-priority solvents are commonly found in various industries and are considered to be ototoxic, neurotoxic and vestibulotoxic (Campo et al., 2009). A combination of solvents with noise can result in significant damage.

The relationship between solvents and noise is complex, particularly as the pathophysiology of SIHL is not fully understood (Mohammadi, Labbafinejad, & Attarchi, 2010). Available research findings regarding the effects of solvents on the auditory system of humans include: damage of sensory cells and nerve endings in the cochlear and in the auditory pathways, damage to the stria vascularis which is the ‘fluid-producing cell layer on the outer wall of the cochlear duct’, damage to the spiral ganglion cells, retro-cochlear damage, vestibular damage and damage to Pillar and Deither cells of the organ of Corti (Campo et al., 2009; Mohammadi et al., 2010).

Combined effects of noise and solvents on the auditory system may have a similar pathophysiology to that of noise-induced hearing loss (NIHL) on the auditory system. Therefore, problems arise in adequately describing the combined effect, as it is not clear as to which specific event leads to auditory dysfunction; that is, does the auditory deficit occur because of noise exposure, solvent exposure or a combination of both? This information is important as data derived can contribute towards policy formulation and amendment of regulations. To date, there has been no shift towards including solvent exposure and monitoring into hearing conservation programmes and medical surveillance programmes.

Three literature reviews were conducted previously regarding the combined effects of solvents and noise on auditory function. Fuente and McPherson (2012) provided a detailed discussion on hearing loss related to various solvents and their interaction with noise. Key findings of the study indicated that there are detrimental effects associated with solvents on the peripheral and central auditory system. The review also found various legislations available globally regarding recommended solvent exposure limits. Augusto, Kulay, and Franco, (2012) conducted a review and concluded that toluene exposure can affect auditory thresholds of workers. The study also found that the audiograms for NIHL present similar toluene-induced hearing loss, thus making it difficult to differentiate between effects of noise and toluene combined and noise only (Augusto et al., 2012). Cary, Clarke, and Delic (1997) conducted a critical review of the literature to determine the effects of exposure to noise and toxic substances combined. The authors concluded that the studies were insufficient to determine any interaction between noise and solvents on hearing (Cary et al., 1997). All reviews were unable to make definitive conclusions regarding the interaction between noise and solvents on hearing. However, several more recent studies with larger sample sizes have since been published, permitting a more detailed review. The aim of the study was, therefore, to conduct a systematic review and meta-analysis to assess the combined effect of solvents and noise on the auditory function of workers within various industrial settings.

## Study objective

The objective was to assess the combined effect of solvents and noise versus noise or solvents only on the auditory function of workers within various industrial settings.

## Methods

### Types of studies

Experimental, cross-sectional studies comparing the audiometric results of groups of workers exposed to noise and solvents versus noise or solvents only.

### Types of participants

The participants were workers who were exposed to a combination of noise and solvents and noise or solvents only within various occupational settings. The workers were of either gender and were aged between 18 and 68 years.

### Types of interventions

Various audiological tests were conducted on workers who were exposed to noise and solvents.

### Types of outcome measures

#### Primary outcome

Hearing loss in workers exposed to solvents only versus both noise and solvents.

#### Secondary outcomes

To identify secondary auditory dysfunctions, which include:

- balance disorders
- upper limit of hearing affected.

### Search methods for identification of studies

#### Electronic searches

To perform a systematic review of the combined effects of solvents and noise on auditory function, a search was conducted for peer-reviewed publications from three different databases. The databases used were Google Scholar, PubMed/Medline and ScienceDirect/Scopus. The following search words were used on all three databases; ‘audiology or solvents or hearing loss or industry’; ‘audiology or solvents or hearing loss’; ‘chemical ototoxicity’; ‘SIHL’; ‘industrial solvents and their effects on hearing’; ‘audiologist or SIHL’, ‘audiology or chemicals or hearing loss or industry’ and ‘xylene or toluene and hearing loss’.

#### Other additional searches

Full-text copies of each of these articles were obtained and read in detail by the review authors. In addition, the references of each article were reviewed to identify possible papers that were missed by the study search. This method of reviewing references of each article was used in order to cross-check results and guarantee that all relevant articles were being used in the review.

## Data collection and analysis

### Identifying studies

Full-text copies of each of these articles were found; all authors of this review paper independently reviewed the articles to ensure that all articles met the inclusion criteria. If one of the review authors were unclear, authors discussed the article’s inclusion/exclusion together. Inclusion criteria included: (1) combined effect of solvents and noise, (2) studies conducted on human beings only and (3) use of audiological tests on participants. Once papers were screened, the abstracts of all records were retrieved to identify obvious exclusions. The reference list of each article was perused to identify possible studies that were missed by the study search. A total of 130 records were initially identified through database searching and a total of 13 studies were included into the review after applying the inclusion criteria. The characteristics of included studies are summarised in Appendix 1 and the characteristics of excluded studies are summarised in Appendix 2.

### Assessment of methodological quality

Heterogeneity was assessed in the selected studies by using the I2 test. The I2 test is used to measure the consistency across studies. This test measures the extent to which the results of the studies were consistent. There was significant heterogeneity evident (high I2).

### Data extraction

The studies were categorised according to: year, country, article title, exposure, objective, design, results, conclusion and references (refer to Appendix 1).

### Data analysis

A meta-analysis was carried out and statistical heterogeneity was assessed. The fixed effect model was used and the odds ratio (OR) was calculated for statistical heterogeneity. An OR is a measure of association between an exposure and an outcome. The OR represents the odds that an outcome will occur, given a particular exposure, compared to the odds of the outcome occurring in the absence of that exposure. Odds ratios are most commonly used in case-control studies; however, they can also be used in cross-sectional and cohort study designs as well (with some modifications and/or assumptions). The pooled estimates for dichotomous outcomes are reported as ORs with 95% CI. The primary comparison was risk of hearing loss in the noise and solvent-exposed group versus noise-only or solvent-only exposed group. Other pair-wise comparisons included solvent-exposed groups versus a control group of no noise or solvent exposure. Heterogeneity of effect sizes was assessed using the I2 statistic (measure of consistency across studies) (Higgins, Thompson, Deeks, & Altman, 2003). As heterogeneity was present (i.e. I2 ≥ 50%), the random effects method was used to estimate a pooled effect size (i.e. OR). All analyses were performed using STATA version 13.0 (StataCorp, 2013). A *p*-value of <0.05 (two-tailed) was considered statistically significant except for the heterogeneity test where a *p*-value cut-off of <0.10 (one-tailed) was used.

## Ethical consideration

Permission to conduct the study was granted by the Social and Human Research Ethics Committee (University of KwaZulu-Natal-HSS/0637/015M).

## Results

A total of 130 peer-reviewed citations were comprehensively reviewed. Of these, 13 papers (3197 workers) were eligible for inclusion. The included studies are summarised in Table 1. The participants’ ages ranged from 18 to 68 years.

**TABLE 1:** Description of number of participants in studies.

Authors	Total	N only (tot)	S only (tot)	N+S (tot)	Control (tot)	N only (cases)	S only (cases)	N+S (cases)	Control (cases)
Barba et al. (2005)	172		82	52	38		20	16	4
Lobato, De Lacerda, Goncalves, and Coifman (2014)	198	42		57	99	7		3	
Hughes and Hunting (2013)	503	148	65	220	70	11	3	12	6
Rizk and Sharaf (2010)	140	50		60	30	9		14	1
Chang et al. (2003)	346	105		131	110	34		89	26
Metwally et al. (2012)	222	70		93	59	44		59	10
Botelho et al. (2009)	152	81		71		13		33	
Mohammadi et al. (2010)	337	173		164		60		113	
Chang, Chen, Lien, and Sung (2006)	174	58		58	58	26		50	3
Ikuharu, Nobuyuki, Hiroichi, and Kazuhisa (2000)	54	19		23	12	5		12	3
Kim et al. (2005)	328	146	18	13	151	25	5	7	9
Prasher, Al-Hajjal, Aylott, and Aksentijevic (2005)	379	153	13	174	39		4	57	2
Schaper, Seeber, and Van Thriel (2008)	192	86		106		53		64	
**Total**	**3197**	**1131**	**178**	**1222**	**666**	**287**	**32**	**529**	**64**

Note: Please see the full reference list of the article, Nakhooda, F., Sartorius, B., & Govender, S.M. (2019). The effects of combined exposure of solvents and noise on auditory function – A systematic review and meta-analysis. *South African Journal of Communication Disorders, 66*(1), a568. https://doi.org/10.4102/sajcd.v66i1.568, for more information.

The studies had the following similarities: all selected studies were conducted on human participants; various audiological tests were conducted across all 13 studies; workers were exposed to a combination of noise and solvents, noise only or solvents only in all the studies; and all studies were conducted in industries with the participants being workers based at the industries. Overview of the meta-analysis results will be presented according to the outcome measures.

Table 1 describes the participants in the studies. The participants were divided into total number of participants recruited and the total number of participants that were used in the study (cases). The participants were further categorised into noise exposure only; solvent exposure only; and combined noise and solvent exposure and control.

## Data synthesis

### Solvents and noise present in studies

Ten articles (77%) contained exposure to noise and a mixture of solvents (methyl ethyl ketone, toluene, xylene and methyl isobutyl ketone), while two studies concentrated on exposure to noise and toluene only (15%) and one study concentrated only on exposure to noise and carbon disulphide (8%).

### Types of audiometric tests used

All 13 studies used pure-tone audiometry testing as part of their test battery. Other tests that were used among the studies included: speech reception testing; speech recognition score; otoscopic examinations; high-frequency audiometry; otoacoustic emissions; auditory brainstem response; videonystagmography (VNG) and posturography; tympanometry; and acoustic reflex threshold.

## Primary outcomes

### Total prevalence of hearing loss

Of the 3197 participants (total of all participants), 35% (*n* =1118) presented with hearing loss as a result of noise exposure only and 35% with solvent exposure only.

**FIGURE 1:**
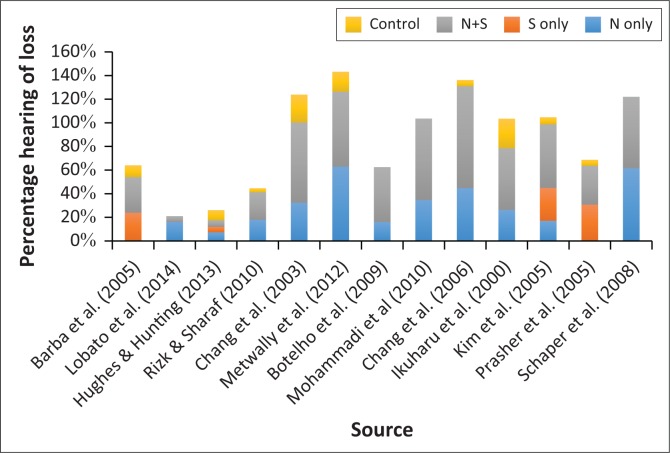
Prevalence of hearing loss among four groups.

Of the 1222 participants (total of all participants exposed to noise and solvents), 43.3% (*n* = 529) presented with auditory pathology as a result of the combined exposure of noise and solvents. The data revealed that the prevalence of hearing loss in the noise and solvent group was significantly (*p* < 0.001) higher than the other groups in 10 out of the 13 studies analysed with pooled OR of 2.754. Many studies did not have a solvent-only group, as solvents often coincide with noise in the working environment. Of the 178 participants (total of all participants exposed to solvents), a total of 32 participants presented with auditory pathology as a result of exposure to solvents only. Figure 1 shows the prevalence of hearing loss among the four groups for each of the included studies.

**FIGURE 2:**
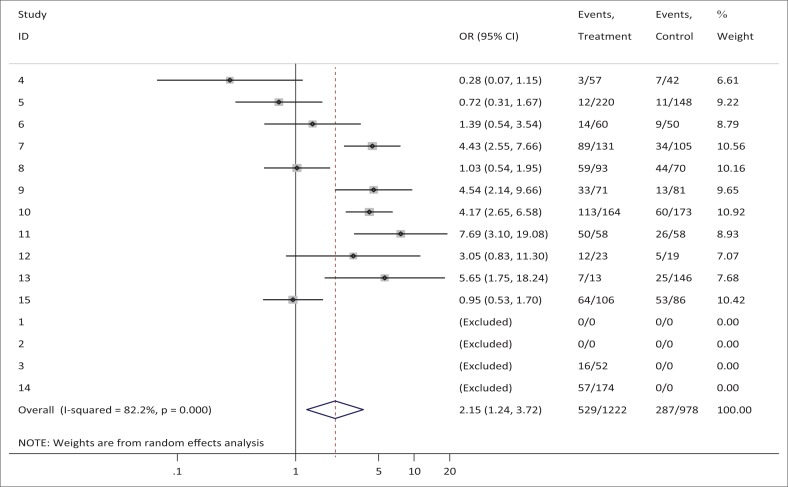
Noise plus solvent versus noise only.

A total of 2285 participants did not present with any form of auditory pathology, despite their exposure to both solvents and noise, or to solvents only, or noise only. The combined estimate of the effects of solvents and noise versus noise only, or solvents only, obtained an OR of 2.146 (see Figure 2). Table 2 and Figure 2 identify that there is a significantly higher pooled odds of hearing loss in a noise- and solvent-exposed group, compared to a solvent-only exposed group (pooled OR = 2.15, 95% CI: 1.24–3.72, *p* = 0.006). The large majority of participants exposed to noise and solvents showed the effects of hearing loss.

**TABLE 2:** Noise plus solvent versus noise only.

Study	OR	95% Conf. interval		% weight
		Lower limit	Upper limit	
Lobato et al. (2014)	0.278	0.067	1.147	6.61
Hughes & Hunting (2013)	0.719	0.308	1.675	9.22
Rizk & Sharaf (2010)	1.386	0.543	3.540	8.79
Chang et al. (2003)	4.425	2.555	7.664	10.56
Metwally et al. (2012)	1.025	0.539	1.950	10.16
Botelho et al. (2009)	4.543	2.136	9.661	9.65
Mohammadi et al. (2010)	4.173	2.647	6.579	10.92
Chang et al. (2006)	7.692	3.102	19.076	8.93
Schaper et al. (2008)	3.055	0.825	11.303	7.07
Kim et al. (2005)	5.647	1.748	18.237	7.68
Ikuharu et al. (2000)	0.949	0.529	1.700	10.42
D+L pooled OR	2.146	1.239	3.718	100.00

Note: Heterogeneity chi-squared = 56.14 (d.f. = 10) *p* = 0.000. I-squared (variation in OR attributable to heterogeneity) = 82.2%. Estimate of between-study variance Tau-squared = 0.6659. Test of OR = 1 : *z* = 2.72 *p* = 0.006. Please see the full reference list of the article, Nakhooda, F., Sartorius, B., & Govender, S.M. (2019). The effects of combined exposure of solvents and noise on auditory function – A systematic review and meta-analysis. *South African Journal of Communication Disorders, 66*(1), a568. https://doi.org/10.4102/sajcd.v66i1.568, for more information.

OR, odds ratio.

### Secondary outcomes

In terms of secondary auditory dysfunctions, only one study reported on the effects of solvents and noise on the upper limit of hearing. Results of the study indicated a reduction of the upper limit of hearing which was the largest in the combined noise and solvent group (Ikuharu et al., 2000). With regard to balance disorders, Prasher et al. (2005) reported on the effects of solvents and noise on hearing and balance in workers. The audiological tests that were used to assess workers’ balance were VNG and posturography. Results revealed that 32% of workers in the solvents and noise group had abnormal posturography and VNG results. It was concluded that the effects of a mixture of solvents on the auditory system appear to occur both at the end organ level, as well as in the nervous pathway (Prasher et al., 2005).

## Discussion

The current review provided evidence of the effects of combined exposure of solvents and noise on the auditory system, revealing a higher prevalence of hearing loss in the noise and solvent group than the other groups in 77% of the studies analysed. Kim et al. (2005) reported in a study conducted on workers within the aviation industry that the prevalence of hearing loss in the noise and solvent group was higher than the other groups (54.9%) and similarly, Chang et al. (2006) reported that the results revealed a higher prevalence of hearing loss in the toluene and noise group (86.2%) when compared to those exposed to noise only (44.8%). Both studies also revealed that the effect of solvents on the auditory system appears to occur both at the end organ level, as well as in the nerve pathway (Mohammadi et al., 2010).

As reported in the studies, long-term exposure to ototoxic solvents can have specific detrimental effects on the auditory system. These effects include: ototoxicity (substances that affect the structure and function of the inner ear and the neural pathways); neurotoxicity (substances that affect the central and peripheral nervous system); and vestibulotoxicity (substances that affect the structure and function of the vestibular organ) (Campo et al., 2009). This triad of complications makes differential diagnosis of SIHL a challenging one, as the symptoms are similar to other auditory pathologies such as NIHL. SIHL coupled with noise exposure makes the relationship even more complex, particularly as very little is known about the pathophysiology of SIHL (Loukzadeh et al., 2014). Thus, research needs to further understand the route of exposure to solvents in order to address this issue, as noise exposure routes have been extensively researched.

Research findings indicate that the vapour particles of solvents are inhaled by workers, which are then absorbed via the respiratory tract (Johnson & Morata, 2010). They can also be absorbed through the skin or exposed wound tissue, after which they translocate into the blood stream and travel through the body and affect cells where they interact with tissue that causes dysfunction in the body and certain organs (Baker, Smith, & Landrigan, 1985, Fuente & McPherson, 2012; Unlu, Kesici, Basturk, Kos, & Yilmaz, 2014; Yah, Iyuke, & Simate, 2011). In addition, once solvents are absorbed into the body via inhalation, the body’s metabolic system transforms the solvents into components that are present in blood and then excreted in urine. Solvents within the body have the potential to interact with various systems within the body, including the auditory system. The key elements with regard to adverse effects on the auditory system depend on the following three issues:

- the toxicity of the solvent
- the rate of absorption
- biotransformation (the process by which the ‘body metabolism transforms solvents into water soluble compounds’). (Lobato et al., 2014; Loukzadeh et al., 2014; Prasher et al., 2005).

Research findings regarding the effects of solvents on the auditory system of humans include: damage of sensory cells and nerve endings in the cochlear and in the auditory pathways; damage to the stria vascularis which is the ‘fluid-producing cell layer on the outer wall of the cochlear duct’; damage to the spiral ganglion cells; retrocochlear damage; vestibular damage; and damage to Pillar and Deither cells of the organ of Corti (Campo et al., 2009; Kim et al., 2005; Mohammadi et al., 2010). Regarding inner ear damage, Campo and Maguin (2007) reported that solvents infiltrate the cochlear and contaminate the tissue as opposed to contaminating the inner ear fluids. The mechanisms involved in auditory damage consist of the solvents travelling via the blood stream and through the stria vascularis, diffusing through the membranes of the cells constituting the outer sulcus and impairing the organ of Corti (Campo & Magun, 2007; Morata, 2003; Sulkowski et al., 2002). Campo, Lataye, Loquet, and Bonnet (2001) in Campo and Maguin (2007) have observed disrupted membranes and concluded that solvents use the outer sulcus as the main route of intoxication to reach the outer hair cells (OHCs). Research has shown that solvents then poison the hair cells, which results in the membranous structures becoming disorganised and causing hair cell death (Campo et al., 2007). In terms of specific OHC damage, the third row of OHCs is thought to be most vulnerable as it is closest to the stria vascularis (Campo et al., 2007) after which the first and second rows are affected (Fuente & McPherson, 2012; Fuente, McPherson, & Hickson, 2013). Furthermore, solvents affect the OHCs in a specific area, that is, the middle turn area, where middle frequencies are located (Choi & Kim, 2014; Sulkowski et al., 2002) and this is different from NIHL where the higher frequencies are generally affected (Johnson & Morata, 2010; Tochetto, Quevedo, and Siqueira, 2013). As discussed by the above mentioned study, individual solvents are reported to cause SIHL (Chang et al. 2003); therefore, it is plausible to assume that a mixture of solvents will have a greater detrimental effect on the auditory system owing to the cumulative effect. From the studies reviewed, participants from 11 out of the 13 studies were exposed to a mixture of solvents and these participants presented with hearing loss.

In terms of audiological tests that were performed, all the studies used pure-tone audiometry testing by assessing the frequency range of 125 Hz–8 kHz, while one study assessed the upper limit of hearing (Ikuharu et al., 2000). In the study by Ikuharu et al. (2000), it was observed that there was an occupational effect of noise and solvents on the upper limit of hearing in workers. The results had shown that noise levels and solvent levels were within occupational exposure limits. There was no significant correlation found between the upper limit of hearing and pure tones and organic solvent concentrations in the working environment. The reduction of the upper limit of hearing was largest in the combined group (Ikuharu et al., 2000). Therefore, it is recommended that high-frequency audiometry be used in the audiological assessment of workers, as it can be used as an early indicator of SIHL (Fuente & McPherson, 2012). Another study included transient and distortion product otoacoustic emissions (OAEs), auditory brainstem potentials (ABR), VNG and posturography (Prasher et al., 2005). The VNG investigations in this study revealed significant abnormalities to the vestibular organs in the group of workers exposed to solvents. More recently, researchers have confirmed that the balance system is affected by solvents. A study in 2011 by Zamyslowska-Szmytke, Politanski, and Sliwinska-Kowalska (2011) discovered that balance abnormalities in solvent-exposed workers indicated subclinical damage, mainly the central part of the vestibular system and body-movement coordination (Zamyslowska-Szmytke, Politanski, & Sliwinska-Kowalska, 2011). The wide variety of the tests indicates that different procedures are needed to detect solvent exposure in different parts of the auditory system. These varieties of tests enable the audiologist to differentiate between cochlear and retrocochlear pathologies and can therefore be used as a guideline for monitoring SIHL. Thus, audiologists need to consider the wide range of effects that solvent exposure could have on the auditory system and include a comprehensive test battery to monitor the affected workers.

### The role of the audiologist regarding solvent-induced hearing loss

The role of the audiologist regarding SIHL is not clearly outlined in the studies mentioned. Solvent-induced hearing loss is a fairly new concern in the field of audiology and presents new challenges for audiologists. Many audiologists are not aware of SIHL, as most research results are published in occupational health journals, which are not typically reviewed by audiologists (Fuente & McPherson, 2006). Audiologists must keep abreast of new knowledge about hazards to hearing to be able to implement programmes for such target groups. They also need to conduct further research within the field of SIHL to expand the literature available, especially with regard to the mechanism and pathophysiology of ototoxic agents, as there is limited research in this area. Results from research conducted can help policymakers establish threshold limit values. Audiologists also have a responsibility to provide information and awareness campaigns to management and stakeholders in order to promote the conservation of hearing among workers. This is particularly important, as certain industries expose their workers to varying levels of solvents depending on the task at hand, with audiologists needing to be aware of the risks to be able to discuss them with management. Audiologists are capable of conducting hearing conservation programmes for workers exposed to solvents. Johnson and Morata (2010) recommend that adjustments need to be made to the hearing conservation programme in combined chemical and noise industries. These adjustments include: taking chemical exposures into account when monitoring air exposures; assessing workers who are exposed to chemicals more regularly; as well as using different methods for controlling workers exposures to chemicals (Johnson & Morata, 2010). Researchers also suggested that the hearing conservation programme needs to include short-interval audiometric evaluations, high-frequency audiometry and efficient hearing protection devices (Mohammadi et al., 2010).

### Future research needs

Future studies need to focus on a longitudinal study design, as this will increase the sample size and thus improve generalisation. One of the main limitations noted in the studies was small sample sizes which meant only minimal conclusions could be drawn from the studies (Chang et al., 2006; Kim et al., 2005; Prasher et al., 2005). In addition, it is worthwhile for research studies to vary the study design from a cross-sectional to a longitudinal design as the studies mentioned used a cross-sectional design and the main limitation of this design is that it cannot establish causal relations (Berg & Latin, 2004). Using a longitudinal design will allow the researcher to assess ototoxic effects effectively, as literature reveals that ototoxic effects occurs over a period of time (Gelfand, 2009). Future research needs to focus on stricter inclusion and exclusion criteria. Researchers did not impose the age limit on their participants in order to control for age effects on hearing. The results could be confounded owing to some workers being above the age of 60 years when typically presbycusis sets in (Ikuharu et al., 2000). This necessitates inclusion of age as a mandatory criterion for future studies. Furthermore, workers within industries may present with variables that may influence the cause and effect relationship (such as factors that affect their hearing, e.g., smoking) regarding noise and solvent exposure on their auditory system, which some studies did not consider, further highlighting the need for stringent inclusion and exclusion participant criteria (Ikuharu et al., 2000). Cumulative dose of exposure is the total dose from conducting repeated air measurements over a period of time (Mohammadi et al., 2010; Nies, 2012). The cumulative dose of exposure is relevant, as it could determine current threshold limits for solvent exposure (Mohammadi et al., 2010). There was a lack of information regarding previous solvent exposure levels of workers and this measure was not calculated for all studies. Further research studies conducted, should attempt to obtain matching sample size numbers in order for appropriate conclusions to be made. Some studies had unmatched numbers across groups, thus only limited conclusions could be drawn from the studies (Prasher et al., 2005). It is recommended that personal dosimetry measurements be conducted for both noise and air measurements, as this will allow for more specific analysis of results per worker. There was a lack of individual samplings (dosimeter and air measurements) of toluene from participants during solvent exposure measurements (Schaper et al., 2008).

## Conclusion

The findings of the systematic review and meta-analysis concluded that there are significantly higher odds of acquiring hearing loss when workers are exposed to a combination of solvents and noise as opposed to solvents only. Globally, there is limited research available on noise and solvent interactions and their effects on hearing. Furthermore, there are only a few comparative studies with varied conclusions, requiring further investigation into the effects of the combined exposure on hearing. Most industries do not control the levels of solvents that they use and do not take into consideration regulations concerning the use of ventilation systems and the provision of masks, gloves or other personal protective equipment which could harm workers, therefore making workers more susceptible to the detrimental effects on the auditory system as a result of combined solvent exposure.

The challenge for the audiologist is that in an occupational environment, as the workers are usually exposed to mixtures of substances, it is not easy to evaluate the effects associated with exposure to a specific chemical. In addition, most threshold limit values are established for a single solvent; however, industries are often composed of several solvents simultaneously. Thus, developed occupational threshold limits are currently based on isolated workplace hazards that are not adequate for protecting workers who may be exposed to multiple solvents in industries coincidently and sequentially. Therefore, recommendations emerging from the studies regarding SIHL for audiologists include:

Prioritising personal solvent monitoring:

- Evaluating personal protective equipment use
- Appropriate recording: Health results of workers should be recorded and checked regularly in order to detect early changes at individual and collective levels
- Risk management measures aimed at reducing exposure to ototoxic substances should be encouraged
- Ototoxicity monitoring that should be made a part of occupational health-screening activities
- Suitable scientific investigations into ototoxic properties should be encouraged such as longitudinal epidemiological studies.

## Appendix 1

**TABLE 1-A1:** Characteristics of included studies.

Article	Variable	Method outlined in the study
Evaluation of combined effect of organic solvents and noise by the upper limit of hearing	Author	Ikuharu et al. (2000)
	Methods	Upper limit of hearing was tested (500 Hz–50 kHz).
		Air conduction testing done.
	Participants	Fifty-four male workers aged between 20 and 68 years.
		Divided into three groups – 23 combined group, 19 noise group, 12 control group.
	Results	Noise levels and solvent levels were within occupational exposure limits. No significant correlation was found between upper limit of hearing and pure tones and organic solvent concentrations in the working environment.
		Reduction of upper limit of hearing was largest in combined group, thus there could be a probable combined effect on hearing even when levels are within limits.
	Outcomes	A probable combined effect of solvents and noise on hearing even when levels were relatively low.
Combined effects of noise and mixed solvents exposure on the hearing function among workers in the aviation industry	Author	Kim et al. (2005)
	Methods	Solvents included methyl ethyl ketone, toluene, xylene and methyl isobutyl ketone.
		PURE-TONE AUDIOMETRY was used.
		14 h rest period before testing.
	Participants	Three hundred and twenty-eight male workers from avionics jobs.
		Exposure to noise (146), solvents (18), noise and solvents (13), none (151).
	Results	Prevalence of hearing loss in noise and solvent group was higher than other groups (54.9%).
	Outcomes	Chronic exposure to mixed solvents had a toxic effect on the auditory system.
Effect of exposure to a mixture of solvents and noise on hearing and balance in aircraft maintenance workers	Author	Prasher et al. (2005)
	Methods	Pure tone audiometry, Otoacoustic Emission’s, Auditory (OAE) brainstem responses, Videonystagmography and posturography were done.
	Participants	Four groups were tested – noise only, solvents only, noise and solvents, none.
	Results	There was a significant effect on PURE-TONE AUDIOMETRY thresholds for noise and for noise and solvent groups.
		OAEs declined with frequency and showed lower DP amplitude with noise compared to noise and solvent group.
		Thirty-two per cent of workers had abnormalities of ABR who were exposed to noise and solvents.
		Thirty-two per cent of workers in solvents and noise group had abnormal posturography results.
		Workers had abnormal results for VNG results in noise and solvent group.
	Outcomes	The effects of a mixture of solvents on the auditory system appears to occur both at the end organ level as well as in the nervous pathway.
Hearing loss in workers exposed to toluene and noise	Author	Chang et al. (2006)
	Methods	Cross-sectional design – one study group and two reference groups.
		Used PT testing.
	Participants	One hundred and seventy four workers at an adhesive materials manufacturing plant.
		Fifty-eight workers exposed to toluene and noise, 58 workers exposed to noise and 58 admin clerks.
	Results	Higher prevalence of hearing loss in toluene and noise group.
		Hearing impairment higher at 1kHz than 2kHz.
		Mean hearing threshold lowest at 6kHz and least effect observed at 2kHz.
	Outcomes	Toluene exacerbates hearing loss in a noisy environment, with the main impact at lower frequencies.
The effects of toluene plus noise on hearing thresholds: An evaluation based on repeated measurements in the German printing industry	Author	Schaper et al. (2008)
	Methods	Four repeated measures over 5 years were done. PURE-TONE AUDIOMETRY was done.
	Participants	Three hundred and thirty three male workers.
	Results	The threshold for developing hearing loss as a result of occupational exposure to toluene plus noise was above the current limit of 50 ppm.
	Outcomes	Owing to missing toluene effects, the conclusion is that the threshold for developing hearing loss as a result of occupational exposure to toluene plus noise might be above the current limit of 50 ppm.
Comparative study of audiometric tests on metallurgical workers exposed to noise only as well as noise associated to the handling of chemical products	Author	Botelho et al. (2009)
	Methods	14 h rest period before testing.
		AC, BC, SRT, SRS was done.
	Participants	One hundred and fifty five workers exposed to noise only 81 (group 1) and also noise and chemicals 71 (group 2).
		Age 18–50 years.
		Working for a period of 3–20 years.
	Results	Greater hearing loss in group 2 (18.3%) than group 1 (6%).
		Chemicals found were styrene, resins and cobalt.
	Outcomes	Group 2 had a proportionally higher hearing loss than group 1.
Combined effects of ototoxic solvents and noise on hearing in automobile plant workers in Iran	Author	Mohammadi et al. (2010)
	Methods	Cross-sectional design.
		Automobile plant.
		PURE-TONE AUDIOMETRY was done.
	Participants	All workers who worked for more than 6 months.
		All male.
		One hundred and sixty four in old paint shop (noise and mixed solvents at high concentration levels). 104 new (noise and mixed solvents at low concentration levels). 173 assembly shop (noise only).
	Results	Solvents found were xylene, toluene, benzene, tetrachloroethylene and acetone.
		High-frequency hearing loss was more common in workers exposed to noise and mixed solvents.
	Outcomes	Combined exposure to mixed solvents and noise can exacerbate hearing loss.
Audiometric findings in petrochemical workers exposed to noise and chemical agents	Author	Barba et al. (2005)
	Methods	The records of environmental noise and solvents measurements and the results of annual audiometry performed by the company were examined.
	Participants	Two groups: group 1 (solvents and noise) and group 2 (noise).
	Results	Despite the low exposure to solvents and a moderate exposure to noise, 45.3% of workers had hearing losses and 29.6% had threshold shifts.
	Outcomes	This study suggests the necessity for reviewing the preventive measurements adopted by the company studied for eliminating the occurrence of hearing losses and standard threshold shift.
Auditory effects of exposure to noise and solvents: A comparative study	Author	Lobato et al. (2014)
	Methods	A transversal retrospective cohort study was performed
	Participants	One hundred and ninety eight workers
		Four groups: noise group, exposed only to noise; the noise and solvents group, exposed to noise and solvents; the noise control group and noise and solvents control group; no exposure.
	Results	The noise group and noise and solvent group had worse thresholds than their respective control groups. Females were less susceptible to noise than males; however, when simultaneously exposed to solvents, hearing was affected in a similar way. The 40- to 49-year-old age group was significantly worse in the auditory thresholds.
	Outcomes	The results observed in this study indicate that simultaneous exposure to noise and solvents can damage the peripheral auditory system.
Evaluation of the effects of exposure to organic solvents and hazardous noise among US Air Force Reserve personnel	Author	Hughes and Hunting (2013)
	Methods	Data were collected retrospectively from existing audiometric examinations, industrial hygiene documentation.
	Participants	Four exposure profiles: Noise with solvents, noise alone, solvents alone and neither noise nor solvents.
		Five hundred and three workers from two Air Force Reserve sites.
		Forty one subjects did not meet the study inclusion criteria.
	Results	Followed for an average of 3.2 years, 9.2% of the study subjects had hearing loss in at least one ear. Increasing age and each year of follow-up time were significantly associated with hearing loss. Low and moderate solvent exposures were not associated with hearing loss.
	Outcomes	Workers who are exposed to increasing levels of noise gradually lose hearing sensitivity over time.
Health hazards among a sample of workers exposed to a combination of noise and organic solvents in a fermentation factory in Egypt	Author	Rizk and Sharaf (2010)
	Methods	All studied sample were subjected to complete medical examination and audiometric examination using pure-tone audiometer.
	Participants	The exposed group consisted of 110 workers in a fermentation plant divided into two groups.
		Group A (50 workers,) exposed to noise only, group B (60 workers) exposed to noise and mixture of organic solvents,
		Control group (group C; 30 workers) were neither exposed to noise nor organic solvents.
	Results	Noise level was comparable in groups A and B but significantly higher than in control work places. Thirty-six per cent of exposed workers suffered from hearing loss versus 3.3 per cent in the control group.
		Hearing loss was significantly higher among group B (24%) than group A (18%). Results showed that both exposed groups had higher hearing loss than normal control.
	Outcomes	Workers exposed to both noise and organic solvents suffered from the highest proportion of hearing loss compared to those exposed to noise alone.
Hearing loss in workers exposed to carbon disulphide and noise	Author	Chang et al. (2003)
	Methods	-
	Participants	One hundred and thirty one men with exposure to noise and CS2 in a viscose rayon plant.
		One hundred and five men in the adhesive tape and electronic industries who were exposed to noise only.
		One hundred and ten men employed in the administrative office of the rayon plant who were exposed to low noise and no CS2.
	Results	Results showed a prevalence of >25 dB hearing loss in rayon workers (67.9%) was much higher than that in administrative workers (23.6%) and in the adhesive tape and electronic industrial workers (32.4%). Hearing loss occurred mainly for speech frequencies of 0.5 kHz, 1 kHz and 2 kHz.
	Outcomes	The study suggests that CS2 exposure enhances human hearing loss in a noisy environment and mainly affects hearing in lower frequencies.
Effect of combined occupational exposure to noise and organic solvents on hearing	Author	Metwally et al. (2012)
	Methods	Questionnaires were given to workers; otoscopic examinations were conducted as well as pure-tone audiometry.
	Participants	Three groups
		Seventy workers exposed to noise only, the second group consisted of 93 workers exposed to organic solvents and noise and the control group included 59 individuals exposed to neither noise nor organic solvents.
	Results	No statistically significant difference between the two exposed groups as regards the duration of exposure. There was a highly statistically significant difference between the two exposed groups as regards the different types of hearing. The difference between the two groups was statistically significant regarding this type of hearing impairment. There was a positive significant correlation between hearing impairment and duration of exposure in the two exposed groups.
	Outcomes	It is recommended that in the case of combined exposure, noise and solvent levels should be lowered than the permissible limits recommended for either alone.

Note: Please see the full reference list of the article, Nakhooda, F., Sartorius, B., & Govender, S.M. (2019). The effects of combined exposure of solvents and noise on auditory function – A systematic review and meta-analysis. *South African Journal of Communication Disorders, 66*(1), a568. https://doi.org/10.4102/sajcd.v66i1.568, for more information.

**TABLE 1-A2:** Characteristics of excluded studies.

Article	Variable	Method outlined in the study
Auditory neuropathy in a patient exposed to xylene: Case report	Author	Draper and Bamiou (2009)
	Methods	-
	Participants	1 adult.
	Results	The patient presented with a gradual deterioration in his ability to hear in difficult acoustic environments and also to hear complex sounds such as music, over a 40-year period.
		His symptoms began following exposure to the solvent xylene, and in the absence of any other risk factor.
		Audiological investigations revealed normal OAEs with absent ABR and absent acoustic reflexes in both ears, consistent with a diagnosis of bilateral auditory neuropathy.
		Central test results were also abnormal, indicating possible involvement of the central auditory pathway.
	Outcomes	This is the first report of retrocochlear hearing loss following xylene exposure.
		The test results may provide some insight into the effect of xylene as an isolated agent on the human auditory pathway.
Audiological findings in individuals exposed to organic solvents: Case studies	Author	Gopal (2008)
	Methods	A battery of audiological tests was administered to all subjects: PURE-TONE AUDIOMETRY, speech and impedance audiometry, OAEs, ABR, MLR, as well as the SCAN-A and R-SPIN tests with low predictability sentence lists.
	Participants	Seven adults – exposed to toluene, xylene and styrene.
		Exposed for at least 3 years.
	Results	All individuals in this study exhibited findings consistent with retrocochlear and/or central abnormality.
		Two of the seven subjects in this study had normal pure-tone thresholds at all frequencies bilaterally, yet showed abnormal retrocochlear/central results on one or more tests.
	Outcomes	The auditory test battery approach used in this study appears to be valuable in evaluating the pathological conditions of the central auditory nervous system (CANS) in solvent-exposed individuals.
Styrene-induced alterations in biomarkers of exposure and effects in the cochlea: Mechanisms of hearing loss	Author	Chen, Chi, Kostyniak, and Henderso (2007)
	Methods	In this study, rats were exposed to styrene at different doses once a day for varying periods.
	Participants	Long-Evans pigmented rats (male, 330 ± 32 g) were used.
	Results	Styrene levels in the cochlear tissues, styrene-induced permanent hearing loss, cochlear disruptions and cell death pathways were determined.
		After 3 weeks of exposure (5 days per week), a dose-dependent permanent hearing loss and a hair cell loss, especially in the mid-frequency region, were observed.
		Deiters cells appeared to be the most vulnerable target of styrene.
	Outcomes	Apoptotic cell death appeared to be the main cell death pathway in the cochlea after styrene exposure.
		In the styrene-induced apoptotic OHCs, histochemical staining detected activated caspases-9 and -8, indicating that both mitochondrial-dependent pathway and death receptor–dependent pathway were involved in the styrene-induced cell death.
Potentiation of noise-induced threshold shifts and hair cell loss by carbon monoxide	Author	Fechter, Young, and Carlisle (1988)
	Methods	Rats received acute exposure to carbon monoxide, noise or both agents concurrently.
		Thresholds were evaluated 2–4 and 6–8 weeks later.
	Participants	Subjects were 16 male Long-Evans hooded rats, weighing between 300 g and 350 g at the start of testing.
	Results	The data showed that carbon monoxide alone does not affect either auditory thresholds or compromise hair cells at the light microscopic level.
		The noise exposure alone produced variable, but quite limited, permanent threshold shifts which were related to the power spectrum of the broad band noise that was employed.
		Hair cell loss was restricted to the basal turn of the cochlea.
		Simultaneous exposure to carbon monoxide and noise-induced large threshold shifts at all frequencies studied, but the effect was greatest at the highest test frequency; an effect not consistent with the noise power spectrum.
		Widespread hair cell loss persisted fully over half of the basilar membrane in the most severely affected rat.
		Outer hair cells appear to be particularly vulnerable.
		Carbon monoxide plus noise did not appear to preferentially disrupt a particular row of outer hair cells.
	Outcomes	These data complement existing evidence that hyperoxia can mitigate against noise-induced injury and reinforce the view that some types of noise-induced damage may result from metabolic insufficiencies.
Ototoxicity of toluene in rats	Author	Sullivan, Rarey, and Conolly (1988)
	Methods	Brainstem auditory evoked responses (BAER) thresholds were recorded from four toluene-treated and four control rats prior to dosing (main experiment) and from all rats after dosing (both experiments).
	Participants	In the preliminary experiment, five male Sprague-Dawley rats were used.
		In the main experiment, eight male Sprague-Dawley rats were used.
	Results	Loss of outer hair cells occurred in all toluene-treated rats in the middle and basal turns of the organ of Corti, with the greatest loss in the third row and progressively less in the second and first rows.
		This loss was more severe in toluene-treated rats that demonstrated elevated BAER thresholds in mid-frequency regions, typically 2–8 kHz.
	Outcomes	These experiments demonstrate that auditory changes are associated with cochlear hair cell loss in toluene-treated rats.
		These ototoxic effects of toluene contrast with those of other known ototoxicants, for example, aminoglycoside antibiotics, in terms of the position of hair cell lesion in the organ of Corti and in the pattern of hair cell loss.

Note: Please see the full reference list of the article, Nakhooda, F., Sartorius, B., & Govender, S.M. (2019). The effects of combined exposure of solvents and noise on auditory function – A systematic review and meta-analysis. *South African Journal of Communication Disorders, 66*(1), a568. https://doi.org/10.4102/sajcd.v66i1.568, for more information.

## References

[CIT0001] Augusto L., Kulay L., & Franco E (2012). Audition and exhibition to toluene – A contribution to the theme. *International Archives of Otorhinolaryngology*, 16, 246–258. 10.7162/S1809-97772012000200015 25991943 PMC4399698

[CIT0002] Baker E. L., Smith T. J., & Landrigan P. J (1985). The neurotoxicity of industrial solvents: A review of the literature. *American Journal of Industrial Medicine*, 8, 207–217. 10.1002/ajim.4700080306 3901738

[CIT0003] Barba M., Jurkiewicz A., Zeigelboim B., Oliveira L., & Belle P (2005). Audiometric findings in petrochemical workers exposed to noise and chemical agents. *Noise and Health*, 7(29), 7–11. 10.4103/1463-1741.31873 17478964

[CIT0004] Berg K., & Latin R (2004). *Essentials of research methods in health, physical education, exercise science and recreation*. Philadelphia, PA: Lippincott Williams & Wilkins.

[CIT0005] Botelho C., Paz A., Goncalves A., & Frota S (2009). Comparative study of audiometrics tests on metallurgical workers exposed to noise only as well as noise associated to the handling of chemical products. *Brazilian Journal of Otorhinolaryngology*, 75(1), 51–57. 10.1016/S1808-8694(15)30831-4 19488560 PMC9442217

[CIT0006] Campo P., & Latave R., Loquet G., & Bonnet P (2001). Styrene-induced hearing loss: A membrane insult. *Hearing Research*, 154(1–2), 170–180. 10.1016/S0378-5955(01)00218-0 11423228

[CIT0007] Campo P., Maguin K., Gabriel S., Moller A., Nies E., Gomez M., et al (2009). *Combined exposure to noise and ototoxic substances*. Luxembourg: Office for Official Publications of European Communities.

[CIT0008] Campo P., & Magun K (2007). Solvent-induced hearing loss: Mechanisms and prevention strategy. *International Journal of Occupational Medicine & Environmental Health*, 20(3), 265–270. 10.2478/v10001-007-0031-3 17932016

[CIT0009] Cary R., Clarke S., & Delic J (1997). Effects of combined exposure to noise and toxic substances: A critical review of the literature. *Annals of Occupational Hygiene*, 41, 455–465. 10.1016/S0003-4878(97)00006-9 9284647

[CIT0010] Chang S., Chen C., Lien C. S., & Sung F. C (2006). Hearing loss in workers exposed to toluene and noise. *Environmental Health Perspectives*, 114, 1283–1286. 10.1289/ehp.8959 16882540 PMC1552019

[CIT0011] Chang S., Shih T., Chou T., Chen C., Chang H., & Sung F (2003). Hearing loss in workers exposed to carbon disulphide and noise. *Environmental Health Perspectives*, 111, 441–447. 10.1289/ehp.6289 PMC1241684 14527841

[CIT0012] Chen G., Chi L., Kostyniak P. J., & Henderson D (2007). Styrene induced alterations in biomarkers of exposure and effects in the Cochlea: Mechanisms of hearing loss. *Toxicological Sciences*, 98(1), 167–177. 10.1093/toxsci/kfm078 17420221

[CIT0013] Choi Y., & Kim K (2014). Noise-induced hearing loss in Korean workers: Co-exposure to organic solvents and heavy metals in nationwide industries. *PLoS One*, 9(5), e97538 10.1371/journal.pone.0097538 24870407 PMC4037174

[CIT0014] Draper T.H., & Bamiou D.E (2009). Auditory neuropathy in a patient exposed to xylene: Case report. *The Journal of Laryngology & Otology*, 123, 462–465. 10.1017/S0022215108002399 18439334

[CIT0015] Fechter L. D., Young J. S., & Carlisle L (1988). Potentiation of noise induced threshold shifts and hair cell loss by carbon monoxide. *Hearing Research*, 34, 39–48. 10.1016/0378-5955(88)90049-4 3403384

[CIT0016] Fuente A., & McPherson B (2006). Organic solvents and hearing loss: The challenge for audiology. *International Journal of Audiology*, 45, 367–381. 10.1080/14992020600753205 16938795

[CIT0017] Fuente A., & McPherson B (2012). *Occupational chemical-induced hearing loss: Hearing loss*. Croatia: InTech.

[CIT0018] Fuente A., McPherson B., & Hickson L (2013). Auditory dysfunction associated with solvent exposure. *BMC Public Health*, 13, 13–39. 10.1186/1471-2458-13-39 23324255 PMC3573910

[CIT0019] Gelfand S (2009). *Essentials of audiology*. New York: Thieme Medical Publishers.

[CIT0020] Gopal K (2008). Audiological findings in individuals exposed to organic solvents: Case studies. *Noise Health*, 10(40), 74–82. 10.4103/1463-1741.44345 19052439

[CIT0021] Higgins J., Thompson S., Deeks J., & Altman D (2003). Measuring inconsistency in meta-analyses. *BMJ*, 327, 557–560. 10.1136/bmj.327.7414.557 12958120 PMC192859

[CIT0022] Hughes H., & Hunting K (2013). Evaluation of the effects of exposure to organic solvents and hazardous noise among US Air Force Reserve personnel. *Noise Health*, 15(67), 379–387. 10.4103/1463-1741.121224 24231416

[CIT0023] Ikuharu M., Nobuyuki M., Hiroichi Y., & Kazuhisa M (2000). Evaluation of combined effect of organic solvents and noise by the upper limit of hearing. *Industrial Health*, 38, 252–257. 10.2486/indhealth.38.252 10812851

[CIT0024] Johnson A. C., & Morata T (2010). Occupational exposure to chemicals and hearing impairment. *Arbeteoch Halsa*, 44, 1–177.

[CIT0025] Kim J., Park H., HA E., Jung T., Paik N., & Yang S (2005). Combined effects of noise and mixed solvents exposure on the hearing function among workers in the aviation industry. *Industrial Health*, 43, 567–573. 10.2486/indhealth.43.567 16100934

[CIT0026] Lobato D., De Lacerda A., Goncalves C., & Coifman H (2014). Auditory effects of exposure to noise and solvents: A comparative study. *International Archives of Otorhinolaryngology*, 18, 136–141. 25992079 10.1055/s-0033-1361083 PMC4296986

[CIT0027] Loukzadeh Z., Shojaoddiny-Ardekani A., Mehrparvar A., Yazdi Z., & Mollasadeghi A (2014). Effect of exposure to a mixture of organic solvents on hearing thresholds in petrochemical workers. *Iranian Journal of Otorhinolaryngology*, 26, 235–243. 25320701 PMC4196447

[CIT0028] Metwally A. M.-A., & El-Tahlawy E (2012). Effect of combined occupational exposure to noise and organic solvents on hearing. *Ind Health*, 28(10), 901–907. 10.1177/0748233711427051 22080036

[CIT0029] Mirzaei R., & Ansari-Moghaddam A (2012). Combined effect of noise and chemical substances on hearing: What is known and future research needs. *Health Scope*, 1, 158–159. 10.5812/jhs.10590

[CIT0030] Mohammadi S., Labbafinejad Y., & Attarchi M (2010). Combined effects of ototoxic solvents and noise on hearing in automobile plant workers in Iran. *Arh Hig Rada Toksikol*, 61, 267–274. 10.2478/10004-1254-61-2010-2013 20860967

[CIT0031] Morata T., & Little M (2002). Suggested guidelines for studying the combined effects of occupational exposure to noise and chemicals on hearing. *Noise Health*, 4, 73–87. 10.1097/01.jom.0000071507.96740.70 12678930

[CIT0032] Morata T. C (2003). Chemical exposure as a risk factor for hearing loss. *JOEM*, 45(7), 676–682. 12855908 10.1097/01.jom.0000071507.96740.70

[CIT0033] Nies E (2012). Ototoxic substances at the workplace: A brief update. Ototoxic substances at work. *Archives of Industrial Hygiene and Toxicology*, 63, 147–152. 10.2478/10004-1254-63-2012-2199 22728796

[CIT0034] Prasher D., Al-Hajjajl H., Aylottl S., & Aksentijevic A (2005). Effect of exposure to a mixture of solvents and noise on hearing and balance in aircraft maintenance workers. *Noise Health*, 7, 31–39. 10.4103/1463-1741.31876 17478967

[CIT0035] Rizk S., & Sharaf N (2010). Health hazards among a sample of workers exposed to a combination of noise and organic solvents in a fermentation factory in Egypt. *Nature and Science*, 8(6), 95–99.

[CIT0036] Schaper M., Seeber A., & Van Thriel C (2008). The effects of toluene plus noise on hearing thresholds: An evaluation based on repeated measures in the German printing industry. *International Journal of Occupational Medicine & Environmental Health*, 21, 191–200. 19042192 10.2478/v10001-008-0030-z

[CIT0037] StataCorp (2013). *[CD-ROM]*. College Station, TX: Statistics.

[CIT0038] Sulkowski W., Kowalska S., Matyja W., Guzek W., Wesolowski W., Szymczak W., et al (2002). Effects of occupational exposure to a mixture of solvents on the inner ear. *International Journal of Occupational Medicine & Environmental Health*, 15, 247–256. 12462452

[CIT0039] Sullivan M. J., Rarey K. E., & Conolly R. B (1988). Ototoxicity of toluene in rats. *Neurotoxicology and Teratology*, 10(6), 525–530. 10.1016/0892-0362(88)90088-8 3244344

[CIT0040] Tochetto T., Quevedo L., & Siqueira M (2013). Hearing conditions of gas stations attendants. *CEFAC*, 15, 1137–1147. 10.1590/S1516-18462012005000107

[CIT0041] Unlu I., Kesici G., Basturk A., Kos M., & Yilmaz O (2014). A comparison of the effects of solvent and noise exposure on hearing, together and separately. *Noise Health*, 16, 410–415. 10.4103/1463-1741.144422 25387537

[CIT0042] Yah C., Iyuke S., & Simate G (2011). A review of nanoparticles toxicity and their routes of exposures. *Iranian Journal of Pharmaceutical Sciences*, 8, 299–314. 22459480

[CIT0043] Zamyslowska-Szmytke E. M., Politanski P., & Sliwinska-Kowalska M. M (2011). Balance system assessment in workers exposed to organic solvent mixture. *International Journal of Occupational Medicine & Environmental Health*, 51(1), 1–10. 10.1097/JOM.0b013e3182143f46 21407091

